# Spontaneous Bowel Evisceration Through Umbilical Hernia Sites in Adult Patients: A Systematic Review of the Literature

**DOI:** 10.3390/clinpract15060099

**Published:** 2025-05-26

**Authors:** Simone Gianazza, Niccolò Grappolini, Marika Morabito, Andrea Palillo, Marta Ripamonti, Davide Inversini

**Affiliations:** 1 Department of General Surgery, Ospedale L Galmarini Tradate—ASST Settelaghi, Piazza Zanaboni 1, 20149 Tradate, Italy; 2 Department of Medicine and Innovation Technology (DiMIT), University of Insubria, 21100 Varese, Italy; ngrappolini@studenti.uninsubria.it (N.G.); mmorabito@studenti.uninsubria.it (M.M.); andrea.palillo@asst-settelaghi.it (A.P.); marta.ripamonti@asst-settelaghi.it (M.R.); davide.inversini@uninsubria.it (D.I.)

**Keywords:** case report, systematic review, umbilical hernia, hernia, small bowel evisceration, evisceration

## Abstract

Background: The literature reports few instances of spontaneous bowel eviscerations through umbilical hernia sites. Spontaneous rupture of the hernia sac is a less common complication, primarily associated with persistent ascites or congenital wall defects. Materials and methods: A systematic review was conducted using the PubMed database—the United States National Library of Medicine, with the search terms “spontaneous bowel evisceration” and “umbilical hernia evisceration”. However, several results were deemed unsuitable for this manuscript. From a total of 185 cases, this review was narrowed down to 9 usable reports. Non-English language cases, duplicates, and cases unrelated to the pathology, including pediatrics, malformations, herniation through other organs, and animal cases, were excluded. Conclusions: Spontaneous evisceration in a hernia is an uncommon yet serious condition. A major risk factor appears to be underlying liver disease with its complications, such as ascites, chronic malnutrition with hypoalbuminemia, and collateral circulation formation. These factors contribute to the susceptibility of the sac and the hernia wall to rupture. However, the limited number of reported cases precludes the establishment of a preferred treatment approach. In the acute phase, the use of prosthetics may be less advisable, but in an elective setting, the cirrhotic patient could be offered repair.

## 1. Introduction

**Figure 1:**
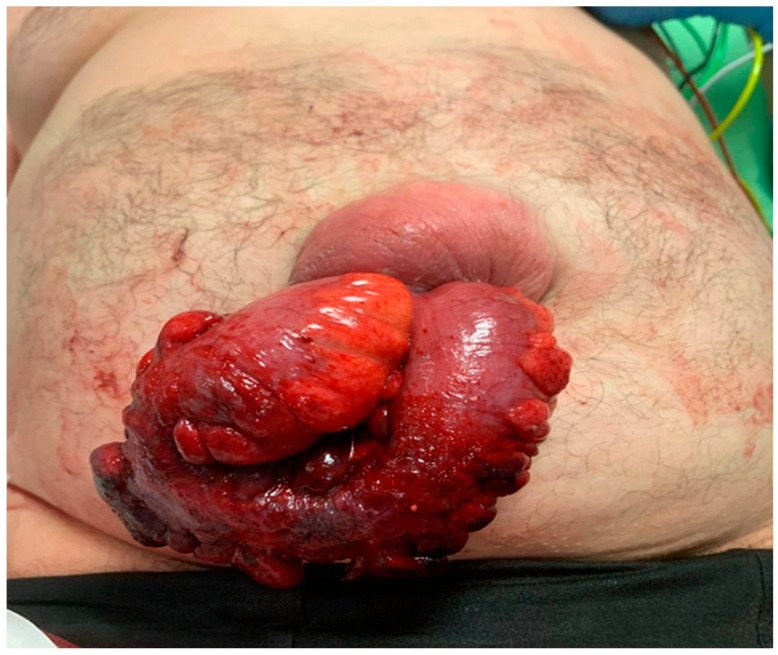
Spontaneous bowel evisceration through an umbilical hernia site in an adult patient.

A hernia is defined as the abnormal protrusion of a viscera or part of a viscera through a defect in the anatomical structure that normally contains it. This common condition, which may be congenital or acquired, affects from 27% to 43% of male patients during their lifetime [1]. Hernias are classified by anatomical location, with umbilical hernias representing a clinically significant subset. According to the European Hernia Society, an umbilical hernia is defined as a ventral midline defect located in a range of 3 cm above or below the umbilicus. Umbilical hernias show a prevalence of 2% in the general population and are acquired in 90% of adult patients. Umbilical hernias are strongly associated with conditions that chronically increase intra-abdominal pressure, including obesity, pregnancy, chronic obstructive pulmonary disease, and ascites. Notably, this pathological condition can be observed in 20 to up to 40% of patients with ascites. The only effective treatment is surgery, and if untreated, it can be prone to complications ranging from mild symptoms to life-threatening emergencies. The clinical consequences of untreated umbilical hernias vary widely. Among the mild but impactful, we must account for the pain and discomfort caused by peritoneal irritation, functional and cosmetic impairment due to the presence of a visible protrusion, and the subocclusive symptoms resulting from an irregularity in gastrointestinal transit relative to the intestinal segment involved in the hernia contents. The spectrum extends to conditions necessitating emergency surgical intervention, such as complete intestinal obstruction and the strangulation and necrosis of the incarcerated bowel. Undoubtedly, one of the most alarming and grave scenarios is the spontaneous rupture of the hernia and the protrusion of its contents through the defect in the abdominal wall [Figure 1] [2]. Spontaneous evisceration is related to an increase in intra-abdominal pressure (e.g., coughing, straining, physical exercise, etc.), causing a sudden rupture of the hernia sac. Management of this complication requires urgent surgical intervention to prevent bowel ischemia, perforation, and sepsis. This manuscript examines the etiology, management strategies, and clinical outcomes of spontaneous evisceration through umbilical hernias, aiming to provide evidence-based insights for managing this rare but life-threatening complication.

## 2. Materials and Methods

A comprehensive systematic review was conducted using the Database PubMed—the United States National Library of Medicine, to identify all available literature on spontaneous bowel evisceration through umbilical hernia sites in adult patients. As the key terms of the search string, the search strategy employed “spontaneous bowel evisceration” AND “evisceration umbilical hernia” to review the literature and capture relevant case reports, case series, and studies about spontaneous bowel evisceration through hernia sites in adult patients. Given the rarity of this condition, no randomized controlled trials or large cohort studies were anticipated; thus, this review focused on synthesizing evidence from smaller, descriptive publications. This systematic review was carried out according to the PRISMA 2020 guidelines, through the completion of the PRISMA Checklist (available as Supplementary Materials [3]), using the PRISMA 2020 Flow Chart to document the study selection process, including screened, excluded, and analyzed records. This review was registered on OSF registries, with the code osf.io/bptxk. The results of the systematic search using the search string “spontaneous bowel evisceration” AND “evisceration umbilical hernia” revealed a limited number of case reports and case series describing this uncommon surgical emergency. This scarcity underscores the exceptional rarity of spontaneous evisceration. The absence of large-scale studies highlights a critical gap in the literature, necessitating a qualitative synthesis of the existing evidence to guide clinical practice.

The aim of this review is to evaluate, in the entirety of the albeit scarce cases existing in the literature, the underlying factors for the condition, the prodromal events for the occurrences, the surgical strategies adopted, and the outcome of the patients who undergo intervention in an urgent/emergency setting. The limited number of case reports and case series suggests that spontaneous bowel evisceration is a rare occurrence and may pose significant diagnostic and management challenges. By consolidating fragmented case-level data, this review seeks to establish preliminary clinical insights, aiding surgeons in early recognition, risk stratification, and optimal management of this life-threatening condition.

## 3. Results

Our systematic review initially identified 185 relevant records published between 1951 and 2024 through PubMed* database searches. Through rigorous screening, we implemented strict exclusion criteria to focus specifically on spontaneous bowel evisceration through umbilical hernias in adult patients. The exclusion process proceeded as follows:

- Congenital anomalies (22 records): Congenital anomalies were excluded, including cases of omphalocele, gastroschisis, and umbilical cord hernias, as these represent fundamentally different embryological defects rather than acquired spontaneous ruptures.
- Pediatric cases (23 records): All childhood presentations were removed to maintain our focus on adult pathology.
- Non-umbilical herniations (64 records): Non-umbilical herniations were removed, including vaginal, rectal, and other atypical herniation sites.
- Irrelevant content (six records): Irrelevant content was removed, including one describing umbilical hernias without evisceration, one detailing parastomal hernias, and four involving animal studies.
- Off-topic publications (24 records): Off-topic publications were removed, including incisional hernia complications (8 records), non-umbilical eviscerations (8 records), post-traumatic cases (3 records), and surgical technique papers (5 records).
- Practical exclusions: Non-English publications (19 records), duplicates (10 records), and records with unavailable data (6 records) were excluded.

**Figure 2:**
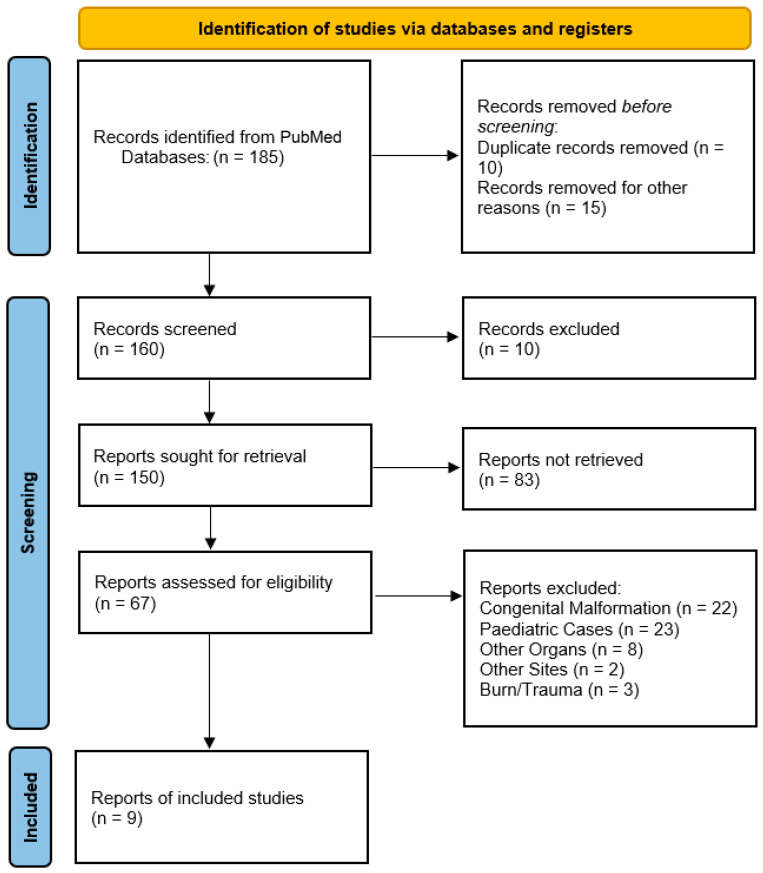
PRISMA flowchart.

This systematic review, conducted using the Database PubMed—United States National Library of Medicine, revealed that spontaneous bowel evisceration through umbilical hernia sites in adult patients is a rare occurrence, as only a limited number of case reports and case series were found describing this surgical emergency. The limited number of cases, along with the diagnostic and management challenges that it poses, suggests that spontaneous bowel evisceration is an uncommon occurrence. After this comprehensive screening, this review identified 9 relevant publications describing 10 cases of spontaneous bowel evisceration through hernia sites, all reported between 2007 and 2024 [Figure 2]. Another recently published case that occurred at our hospital was added to this review. Recently, at our institute (Ospedale di Circolo e Fondazione Macchi—Varese, Italy), a 53-year-old man, without a previous history of hepatic disease, acceded with evisceration of the small bowel through an umbilical hernia caused by sudden coughing and was successfully treated with bowel resection (due to the sub-ischemic features of the ileal loop), an direct repair of the abdominal fascia [Table 1].

Given the scarcity of reported cases in the literature (only 11 total cases, including ours) and the inability to conduct an adequate statistical analysis, the individual cases were considered a source of learning and are discussed as such for this qualitative review. The individual articles were analyzed to extract the common data (demographic characteristics, predisposing factors, clinical presentation, surgical management, outcomes) present in each case report for the purpose of constructing a summary table that includes the comparable parameters.

## 4. Discussion

Congenital umbilical hernias are one of the most common conditions seen in infants and children, with an estimated prevalence of 10–20% [4]. These hernias typically result from a failure of the rectus abdominis muscles to approximate in the midline following the return of the midgut into the peritoneal cavity, leaving a persistent defect in the linea alba. This condition is related to prematurity (significantly higher in preterm infants) or associated with several syndromes, such as trisomy 21 (Down syndrome), Beckwith-Wiedemann syndrome, and hypothyroidism [4,5]. They are normally asymptomatic and spontaneously regress with spontaneous closure by age 4–5 years. While generally benign, rare complications can occur.

Evisceration is a rare complication in pediatric patients. Wendy L. et al. described 19 cases of spontaneous evisceration in pediatric umbilical hernia over 50 years [6]. This means that the risk of this complication is small, considering the frequency of this pathology. Several factors precipitate hernia rupture, such as the age of the infant or child, the defect size (larger than 1.5 cm in diameter), umbilical sepsis or ulceration of the skin, and any condition that suddenly increases the intra-abdominal pressure, i.e., vigorous crying, respiratory infection, intussusception [7]. Adequate treatment of congenital hernias has a favorable outcome in contrast to adult cases. In contrast to pediatric cases, umbilical hernia in adults is a very frequent wall defect [1]. This is related to the progressive weakening of the umbilical ring associated with an acute or chronic rise in intra-abdominal pressure. One of the most important pathological causes of abdominal pressure increase is ascites, the risk of which is 20% [8,9,10,11,12,13]. Obesity (BMI > 30 increases the risk 3-fold), visceromegaly, pregnancy (with multiparity as an independent risk factor), large cysts, and neoplastic lesions are the other common causes of umbilical hernias [14,15,16,17]. The risk of spontaneous hernia rupture with evisceration is infrequent. In a literature review without time selection limits, the first case described was in 1951. We found only 10 published cases in the last 24 years of adult patients with wall defects as the site of evisceration, in addition to the unpublished case that occurred recently at our hospital. Of these, five were women and six were men. In those cases, the umbilicus becomes a site affected by evisceration because the continuity of the linea alba is interrupted, and, for this reason, it represents an area of weakness. The umbilicus, by its nature, represents a point of weakness in the anterior abdominal wall, as a derivative of the stump of the umbilical cord. Being located medially between the two rectus muscles, it causes the anatomical and mechanical interruption of their muscular aponeurosis, thus creating an area of lower resistance to abdominal pressure. Tensile forces, therefore, act on this site, which, in addition to protein deficiency, sarcopenia, obesity, pregnancy, and chronic disease (e.g., diabetes, collagen disorders, etc.) that decrease the resistance of the muscles and the abdominal wall, cause abnormal decussations of the aponeurotic fibers with the consequent onset of hernial defects. The age of the patients ranged from 23 to 81 years old. Five cases reported a growth of the hernia in the last period [9,10,13,18,19]; in the other cases, there was a sudden onset. In six cases, evisceration was preliminarily associated with leakage of ascitic fluid [10,11,12,20,21]. In 8 out of 11 cases, the event occurred in patients with chronic liver failure, with cirrhosis on an alcoholic basis, in 4 out of 8 [10,11,12,20]; hepatitis, in 3 out of 8 [9,10,21]; or combined alcoholic and hepatitis, in 1 out of 8 [13]. These cases were always associated with an important ascitic component. From the cases described, it seems well established that the greatest risk factor in spontaneous evisceration related to a viral or alcoholic hepatopathy [9,10,11,12] is degeneration into cirrhosis, associated with eight out of the eight cases with ascites. The ascites led to a chronic elevation in intra-abdominal pressure. Body position and patient activity cause a further increase in the intra-abdominal pressure, which can overwhelm the strength of the anterior abdominal wall layers. Skin ulceration and the rapid increase in the size of the hernia are signals of impending rupture. Chronic cutaneous ulcerations and their resulting inflammatory response, in addition to the picture of ascites, can cause a reduction in the tone and thickness of the anterior abdominal wall. This can lead to liquid spillage from the hernia defect, a sign of possible impending rupture [17]. In addition to these conditions, acute and subacute changes in intra-abdominal pressure, such as vomiting and coughing, led to hernia rupture. Even in our case, the patient complained about a rupture of the abdominal wall defect after a sudden cough, but in contrast with the literature, the patient had no previous medical history of hepatitis infection, even without cirrhosis and ascites [22]. Likely, a chronic increase in intra-abdominal pressure caused by obesity and the sudden cough led to the umbilical hernia rupture. In this review, the herniated viscera were the ileum in six cases [12,18,19,20,21] and in the omentum in five cases [9,10,11,13]. Resection was necessary in two cases in the literature [13,19], as in our patient, because of the ischemic findings of the herniated viscera. Evisceration is a life-threatening emergency because of the risk of perforation, ischemic complications of the visceral herniation, or septic complications. In an emergency setting, direct suture repair was preferred. Abdulqader M. et al. [9] declared that urgent umbilical herniorrhaphy without mesh or primary closure is the preferred intervention in cirrhotic patients presenting with an umbilical hernia rupture since it has been shown to reduce the mortality to 6–20% [23]. In 10 cases, direct repair of the hernia was required to address the high risk of infection. A lot of studies underline that the placement of a prosthesis reduces the rate of hernia recurrence [8,24]; however, in these emergency settings, only one case of mesh repair was found [9]. In that case, the patient was young, clinically stable without signs of infection, and without the necessity of bowel resection. The authors placed polypropylene mesh over the posterior rectus sheath and below the rectus. The sublay technique can be used as an elective in an emergency, with a good outcome. Despite this, there remains a reluctance to place a prosthetic mesh in a certainly contaminated setting of evisceration. This attitude can be attributed to the fear of the occurrence of infectious complications, considering that the use of a mesh may result in a higher incidence of infection, at least in the short term, especially in patients with pre-existing pathological conditions predisposing to possible risks. This eventuality would be avoided with a direct repair, which, however, determines a higher recidivism rate. Additionally, especially when involving obese patients, the development of a large retromuscular space for the placement of a sublay mesh could further burden the eventual complications dictated by a likely contaminated setting. However, the placement of a prosthetic mesh could be considered if the extensive loss of tissues following the intervention does not allow for reconstruction with the layers of the abdominal wall alone, making it, therefore, necessary to reconstruct the defect [9,25]. Between the described cases, the mortality after surgery reached a rate of 30%, and all the deceased patients showed chronic ascites and end-stage disease [26]. All the deaths were related to underlying clinical conditions, where one patient passed away because of acute renal failure and the other because of massive hematemesis in esophageal varices. The third deceased patient died subsequently of spontaneous bacterial peritonitis. In eight cases, however, an uneventful discharge was described.

This qualitative review provides clinical recommendations regarding risk stratification through monitoring for rapid hernia growth and the presence of ulcerations.

As a result, it is necessary to consider elective repair in high-risk cirrhotic patients with mesh placement when feasible. In emergency cases, primary repair remains the gold standard. Multidisciplinary evaluation is essential for the cirrhotic patient to manage ascites and malnutrition.

Future research on this topic should focus on identifying predictive risk factors, comparing repair techniques, and evaluating long-term outcomes, including through the establishment of an international registry.

## 5. Conclusions

Spontaneous bowel evisceration through an umbilical hernia represents a rare but catastrophic complication. The cases are usually associated with refractory ascites in cirrhotic patients with end-stage liver disease. The surgical management of these cases is associated with a substantial mortality rate of up to 30% [26], reflecting the physiological frailty of this patient population. On the contrary, as only a few reports are described in the literature, our case presented a spontaneous evisceration of the bowel in a non-cirrhotic patient. This might be one of the reasons why he had a good outcome, suggesting that the absence of hepatic dysfunction may confer prognostic benefits. Cirrhosis results in several complications that make the sac and herniary port more fragile and more susceptible to rupture. The predisposition of cirrhotic patients to spontaneous rupture stems from multiple interrelated factors. This includes ascites, which increases intra-abdominal pressure that progressively weakens the hernia sac and surrounding tissues. The protein–calorie malnutrition with hypoalbuminemia, from which these patients suffer, promotes impaired wound healing and progressive sarcopenia (reducing abdominal wall integrity). Moreover, the progression of portal hypertension often results in the recanalization of the umbilical vein and the formation of collateral circles, such as caput medusae. These promote an enlargement in the umbilical defect and the appearance of a hernia. These pathological changes create a favorable situation for spontaneous rupture, particularly during episodes of transient pressure elevation, such as coughing, vomiting, or straining. Given the rarity of this condition, no standardized treatment protocol exists. However, analysis of available cases suggests an emergency management with immediate fluid resuscitation and broad-spectrum antibiotics. Regarding the surgical approach, patients should be treated with exploratory laparotomy to assess bowel viability, performing resection if necrotic, and primary fascial repair. Primary fascial repair is preferred in contaminated cases, with mesh avoidance in acute settings, because of infection risk. However, if the clinical conditions of the patient allow, and the risk of infection is low, it is possible to place a mesh using different techniques. Elective hernia repair with mesh reinforcement could be recommended in cirrhotic patients with well-controlled ascites, to reduce the risk of complications, and in non-cirrhotic patients. Techniques may include retrorectus mesh placement or onlay reinforcement. These approaches are useful even if the wall defect is symptom-free, to protect patients from the possible complications previously described.

Regarding the emergency setting, this study shows evident limitations dictated by the extremely small number of cases reported in the literature and the absence of a unitary guideline in its specific treatment, with heterogeneous management approaches across reported cases, in the absence of controlled comparative studies. Nevertheless, a critical review of the available evidence can provide valuable insights to guide the clinician in the management of such a condition. Our study, therefore, positions itself as the first comprehensive synthesis to re-examine all the published cases in their entirety, granting practical insights for future research and constituting a foundation for future research. Further and future studies will be necessary to expand the knowledge of the subject and define an optimal and shared therapeutic strategy.

Prospective multicenter registries could help to establish risk stratification models, refine optimal timing for mesh use, and develop evidence-based management algorithms.

## Figures and Tables

**Table 1:** Records and patient data according to the results of this review.

Author	Age	Sex	Growth	Ulcerations	Leakage	Cirrhosis	Ascites	Viscera	Repair	Resection	Outcome
Arora	42	F	Y	N	Y	Y (Hepatic)	Y	Omentum	Suture	N	Exitus
Arora	40	M	N	Y	Y	Y (Alcoholic)	Y	Omentum	Suture	N	Exitus
Albeladi	23	F	Y	Y	N	Y (Hepatic)	Y	Omentum	Mesh	N	Discharged
Bolivar R	46	F	Y	Y	N	N	N	Ileus	Suture	N	Discharged
Good	81	M	N	Y	Y	Y (Alcoholic)	Y	Omentum	Suture	N	Discharged
Choo	41	M	N	Y	Y	Y (Alcoholic)	Y	Ileus	Suture	N	Discharged
Ginsburg	45	M	Y	Y	N	Y (Both)	Y	Omentum	Suture	Y	Exitus
Ahmed	28	F	Y	Y	N	N	N	Ileus	Suture	Y	Discharged
Iizuka	42	F	N	Y	Y	Y (Alcoholic)	Y	Ileus	Suture	N	Discharged
Ogu	50	M	N	N	Y	Y (Hepatic)	Y	Ileus	Suture	N	Discharged
Grappolini	53	M	N	Y	N	N	N	Ileus	Suture	Y	Discharged

F, female; M, male; Y, yes; N, no.

## Data Availability

The original contributions presented in this study are included in this article; further inquiries can be directed to the corresponding author.

## Supplementary Materials

The following supporting information can be downloaded at: https://www.mdpi.com/article/10.3390/clinpract15060099/s1, Table S1: PRISMA 2020 checklist.

## References

[1] Kingsnorth A., LeBlanc K. (2003). Hernias: Inguinal e incisional. Lancet.

[2] Gupta R.K., Sah S., Agrawal S.C. (2011). Spontaneous rupture of incisional hernia: A rare cause of a life-threatening complication. BMJ Case Rep..

[3] Page M.J., McKenzie J.E., Bossuyt P.M., Boutron I., Hoffmann T.C., Mulrow C.D., Shamseer L., Tetzlaff J.M., Akl E.A., Brennan S.E. (2021). The PRISMA 2020 statement: An updated guideline for reporting systematic reviews. BMJ.

[4] Garcia V.F., Ashcraft K.W. (2000). Umbilical and other abdominal wall hernias. Paediatric Surgery.

[5] Ciley R.E., Krummel T.M., O’Neil J.A., Rowe M.I., Grosfield J.L. (1998). Disorders of the umbilicus. Paediatric Surgery.

[6] Thomson W.L., Wood R.J., Millar A.J.W. (2012). A literature review of spontaneous evisceration in paediatric umbilical. Pediatr. Surg. Int..

[7] Weik J., Moores D. (2005). An unusual case of umbilical hernia rupture with evisceration. J. Paediatr. Surg..

[8] Eker H.H., Van Ramshorst G.H., De Goede B. (2011). A prospective study on elective umbilical hernia repair in patients with liver cirrhosis and ascites. Surgery.

[9] Albeladi A.M., Odeh A.M., AlAli A.H., Alkhars A.M., Buhlaigah A.M., Alghadeer H.A. (2021). AlGhadeer Spontaneous Umbilical Hernia Rupture Associated With Omentum Evisceration in a Patient With Advanced Hepatic Cirrhosis and Refractory Ascites. Cureus.

[10] Arora E., Gandhi S., Bhandarwar A., Quraishi A.H.M., Wagh A., Tandur A., Wakle D. (2018). Umbilical Hernia with Evisceration. Two Cases and a Review of the Literature. J. Emerg. Med..

[11] Good D.W., Royds J.E., Smith M.J., Neary P.C., Eguare E. (2011). Umbilical hernia rupture with evisceration of omentum from massive ascites: A case report. J. Med. Case Rep..

[12] Choo K., McElroy S. (2008). Spontaneous bowel evisceration in a patient with alcoholic cirrosi and an umbilical hernia. J. Emerg. Med..

[13] Ginsburg Y., Sharma Adhi N. (2006). Spontaneous rupture of an umbilical hernia with evisceration. J. Emerg. Med..

[14] Sugerman H., Windsor A., Bessos M., Wolfe L. (1997). Intra-abdominal pressure, sagittal abdominal diameter and obesity comorbidity. J. Intern. Med..

[15] Wilson A., Longhi J., Goldman C., McNatt S. (2010). Intra-abdominal pressure and the morbidly obese patients: The effect of body mass index. J. Trauma Acute Care Surg..

[16] Twardowski Z.J., Tully R.J., Ersoy F.F., Dedhia N.M. (1990). Computerized tomography with and without intraperitoneal contrast for determination of intraabdominal fluid distribution and diagnosis of complications in peritoneal dialysis patients. ASAIO J..

[17] Lemmer J.H., Strodel W.E., Knol J.A., Eckhauser F.E. (1983). Management of spontaneous umbilical hernia disruption in the cirrhotic patient. Ann. Surg..

[18] Bolívar-Rodríguez M.A., Magaña-Zavala P.A., Pamanes-Lozano A., Fragoza-Sánchez E. (2021). Evisceration due to spontaneous rupture of umbilical hernia in adult. Cir. Esp..

[19] Ahmed A., Stephen G., Ukwenya Y. (2011). Spontaneous rupture of umbilical hernia in pregnancy: A case report. Oman Med. J..

[20] Yuki I., Mayu H., Yusuke S., Maki T., Kazuhiro S., Yuichi H. (2022). Intestinal evisceration and Staphylococcus aureus bacteremia due to ruptured umbilical hernia in a patient with liver cirrhosis: A case report and literature review. Oxf. Med. Case Rep..

[21] Ogu U.S., Valko J., Wilhelm J., Dy V. (2013). Spontaneous evisceration of bowel through an umbilical hernia in a patient with refractory ascites. J. Surg. Case Rep..

[22] Grappolini N., Zanchetta M., Inversini D., Ietto G. (2024). Spontaneous bowel evisceration through umbilical hernia in an adult non-cirrhotic patient. BMJ Case Rep..

[23] Chatzizacharias N.A., Bradley J.A., Harper S., Butler A., Jah A., Huguet E. (2015). Successful surgical management of ruptured umbilical hernias in cirrhotic patients. World J. Gastroenterol..

[24] McKay A., Dixon E., Bathe O., Sutherland F. (2009). Umbilical hernia repair in the presence of cirrhosis and ascites: Results of a survey and review of the literature. Hernia.

[25] Den Hartog D., Dur A.H., Tuinebreijer W.E., Kreis R.W. (2011). Open surgical procedures for Incisional hernias. Cochrane Database Syst. Rev..

[26] Arowojolu O.A., Mitchell O.J.L., Liu S. (2017). FLOOD Syndrome: Not Your Average Paracentesis.

